# Fulminate mycoplasma infection and severe cutaneous vasculitis: an unusual presentation of complete MBL deficiency

**DOI:** 10.1186/1546-0096-9-S1-P211

**Published:** 2011-09-14

**Authors:** Despoina Maritsi, M Al-Obadi, Alexandra Tabor, PA Brogan

**Affiliations:** 1 Great Ormond Street Hospital, LONDON, United Kingdom

## 

A 12 year old Caucasian female presented with malaise, pyrexia and painful bullous hemorrhagic skin lesions on both upper and lower extremities. She was the second child of a healthy unrelated Caucasian couple and her past medical history was unremarkable. Laboratory tests showed raised inflammatory markers and chest radiograph showed a left lower lobe collapse. She was started on intravenous broad spectrum antibiotics and steroids, with a provisional diagnosis of vasculitis secondary to infection. Autoimmune tests (including ANCA) were negative and she had no evidence of systemic vasculitis. Given the extent of the cutaneous lesions, tests looking at the immune system and for an infection precipitant were performed. Fluid aspirate from the lesion was sterile and skin biopsy showed non specific inflammation. Mycoplasma serology titers were extremely high 1:5200 and the patient had complete mannose binding lectin deficiency (MBL). She was treated with macrolides and was started on chemoprophylaxis.

Mannose binding lectin (MBL) is a serum protein believed to play an important role in the innate immune response. Defective MBL production is regarded as the most common immune deficiency in the general population, affecting approximately 5-7% of individuals. Severe MBL usually presents in the early infancy or in early childhood. Late presentation with severe clinical features such as this is not well documented. Patients with primary antibody deficiencies are susceptible to mycoplasma infections (mainly arthritis) due to defective function on the mannose binding lectin pathway. Conversely, mycoplasma is a common pathogen in the paediatric population whilst erythema multiforme associated with mycoplasma infection is a rare presentation. This case is first report in literature where complete MBL deficiency presented in late childhood with severe mycoplasma infection related severe cutaneous vasculitis and no previous history of recurrent infections.

